# Efficacy and Safety of Stereotactic Body Radiation Therapy Modalities for >5 cm Advanced Unresectable Hepatocellular Carcinoma: A Network Meta-Analysis

**DOI:** 10.3390/cancers18060988

**Published:** 2026-03-18

**Authors:** Henry W. C. Leung, Shyh-Yau Wang, John Hang Leung, Yun-Sheng Tai, Agnes L. F. Chan

**Affiliations:** 1 Department of Radiation Oncology, An-Nan Hospital, China Medical University, Tainan 709, Taiwan; 070506@tool.caaumed.org.tw; 2 Department of Radiology, An-Nan Hospital, China Medical University, Tainan 709, Taiwan; 3 To Be Better One Aesthetic Clinic, Taipei City 100, Taiwan; 4 Department of Breast Surgery, An-Nan Hospital, China Medical University, Tainan 709, Taiwan; 5 Department of Research, An-Nan Hospital, China Medical University, Tainan 709, Taiwan

**Keywords:** tumor microenvironment (TME), stereotactic body radiotherapy (SBRT), transcatheter arterial chemoembolization (TACE), inoperable aHCC, immunotherapy-based combinations

## Abstract

**Objective**: Radiotherapy remodels the tumor microenvironment (TME) and may enhance the efficacy of immunotherapy in cancer treatment, particularly in patients with large, unresectable hepatocellular carcinoma (HCC) complicated by portal vein tumor thrombus (PVTT). Because of these unique effects, a growing body of research has found that stereotactic body radiation therapy (SBRT) combined with transcatheter arterial chemoembolization (TACE) or programmed death protein 1 (PD-1) inhibitors has a synergistic impact on unresectable advanced hepatocellular carcinomas (HCCs) larger than 5 cm in diameter. We aim to explore the efficacy of these treatment modalities through a network meta-analysis (NMA). **Methods and Analysis**: We evaluated the efficacy and safety of different SBRT-based treatment modalities for large advanced HCCs with PVTT (tumor diameter ≥ 5 cm), with primary endpoints including overall survival (OS), progression-free survival (PFS), and grade 3–4 severe adverse events (SAEs). **Results:** Eighteen studies comprising 2303 patients were included. SBRT combined with transcatheter arterial chemoembolization (SBRT + TACE) demonstrated significantly superior overall survival compared with other monotherapy or combination strategies. Most other treatment regimens showed comparable PFS outcomes. Notably, SBRT alone and SBRT combined with PD 1 inhibitors (SBRT + PD 1) were associated with significantly lower incidences of severe adverse events compared with other treatment modalities; all of these reported SAEs were manageable with appropriate clinical intervention. **Conclusions**: For patients with large (≥5 cm) advanced HCC with PVTT, SBRT combined with TACE was associated with superior OS and PFS compared with other treatment strategies. These findings suggest potential synergistic interactions between SBRT and TACE or immunotherapy. Further high-quality prospective trials are warranted to validate these observations and clarify the underlying molecular mechanisms. Our results provide evidence to inform therapeutic decision-making in advanced HCC.

## Simple Summary

Radiation therapy can remodel the tumor microenvironment (TME) and may enhance the efficacy of immunotherapy in cancer treatment. Stereotactic radiotherapy (SBRT) can precisely deliver high doses of ablation radiation within a limited number of irradiation events, thereby achieving effective local tumor control and unique immunomodulatory effects. In this study, we observed that SBRT combined with transcatheter arterial chemoembolization (SBRT + TACE) significantly improved overall survival compared with other mono- or combination therapies. Both SBRT and TACE may have mechanisms that promote the release of tumor-associated antigens and risk-related molecular patterns into the microenvironment, potentially enhancing T-cell activation, effects may counteract the immunosuppressive TME specific to advanced hepatocellular carcinoma (HCC).

## 1. Introduction

Two decades ago, the hypothesis that the “abscopal effect” may be mediated by immune-related sequelae of ionizing radiation was proposed [1], and so increasing attention has since been directed toward the complex interplay between radiotherapy and the tumor microenvironment (TME). Radiotherapy (RT) not only induces direct tumor cell death but also promotes the release of pro-inflammatory mediators, enhances antigen presentation, and facilitates immune cell infiltration, thereby remodeling the TME [2]. These immunomodulatory effects may shift the balance between immune activation and suppression within the tumor milieu.

Stereotactic body radiotherapy (SBRT) enables the precise delivery of high ablative radiation doses in a limited number of fractions, an approach which has demonstrated potent local tumor control and distinct immunomodulatory properties. Consequently, combining SBRT with immunotherapy has emerged as a promising strategy in several malignancies, including metastatic renal cell carcinoma and non-small-cell lung cancer [3,4,5,6]. In hepatocellular carcinoma (HCC), the immunomodulatory effects of SBRT may enhance the antitumor efficacy of immune checkpoint inhibitors (ICIs), particularly the Programmed Cell Death Protein 1 (PD-1) blockade [7,8,9].

Phase II clinical trials have reported encouraging outcomes of SBRT combined with ICIs in patients with unresectable HCC, with objective response rates of 52.4% and 1-year overall survival rates approaching 59.9% [10]. Several clinical trials and real-world studies have further demonstrated the therapeutic potential of SBRT–ICI combinations in advanced HCC with portal vein tumor thrombus (PVTT) and tumor diameters ≥ 5 cm [11,12,13], consistently suggesting improved survival benefits compared with monotherapy.

In addition to immunotherapy, SBRT has also been combined with transcatheter arterial chemoembolization (TACE) and other locoregional or systemic treatments. For advanced HCC with tumors larger than 5 cm, increasing evidence suggests that SBRT-based combination strategies may improve survival outcomes, and these approaches are referenced in the 2025 NCCN guidelines [14].

Despite growing clinical evidence, direct comparisons among different SBRT-based combination modalities remain limited. To our knowledge, no previous network meta-analysis has comprehensively synthesized the available evidence to compare the efficacy and safety of SBRT-related treatment strategies in advanced HCC with PVTT and tumor diameters ≥ 5 cm; therefore, in the present study, we aim to evaluate and rank SBRT-based modalities using a network meta-analytic approach and to explore their potential mechanistic and clinical implications.

## 2. Methods

### 2.1. Search Strategy and Study Design

This network meta-analysis was conducted in accordance with the Preferred Reporting Items for Systematic Reviews and Meta-Analyses (PRISMA) guidelines [15]. The literature search covered studies published between January 2016 and June 2025, and the study protocol was not prospectively registered.

A comprehensive search was performed in PubMed, Embase, and the Cochrane Central Register of Controlled Trials (CENTRAL) using the detailed strategy provided in Supplementary Table S1. Keywords and Medical Subject Headings (MeSH) included combinations of the following terms: “hepatocellular carcinoma,” “tumor diameter ≥ 5 cm,” “portal vein tumor thrombus,” “stereotactic body radiotherapy,” “SBRT,” “programmed cell death-1 inhibitor,” and “clinical trials.” Searches were limited to articles published in English and reference lists of relevant articles were manually screened to identify additional eligible studies. The study selection process is illustrated in the PRISMA flow diagram (Supplementary Figure S1).

### 2.2. Eligibility Criteria and Data Extraction

**Inclusion Criteria**

Studies were included if they met the following criteria: (1) prospective or retrospective clinical studies evaluating SBRT-based treatment strategies in hepatocellular carcinoma with tumor diameter ≥ 5 cm and portal vein tumor thrombus (PVTT); (2) comparisons involving SBRT alone or combined with PD-1 inhibitors, transcatheter arterial chemoembolization (TACE), or other therapeutic modalities; (3) reported clinical outcomes including overall survival (OS), progression-free survival (PFS), or grade 3–4 severe adverse events (SAEs); and (4) provided sufficient data to estimate effect sizes (odds or hazard ratios with corresponding 95% confidence intervals).

**Exclusion Criteria**

Studies were excluded if they met the following exclusion criteria: (1) were reviews, editorials, comments, conference abstracts without full data, or duplicate publications; (2) did not report extractable OS or PFS data; and (3) contained overlapping populations (in which case, the most recent or most comprehensive study was included).

Study selection was performed independently by two investigators (SY and AC), with disagreements resolved through discussion and a third reviewer (LH) consulted when necessary. Data extracted included study characteristics (author, year, design), patient demographics, treatment regimens, follow-up duration, and outcome measures (OS, PFS, SAEs).

### 2.3. Quality Assessment

#### 2.3.1. Risk of Bias Assessment

Among the included studies, 17 were non-randomized cohort studies. Risk of bias for the non-randomized studies was assessed independently by two reviewers using the Cochrane ROBINS-I (Risk Of Bias In Non-randomized Studies of Interventions) tool [16], which evaluates bias across seven domains: (1) due to confounding, (2) in the selection of participants, (3) in the classification of interventions, (4) due to deviations from intended interventions, (5) due to missing data, (6) in the measurement of outcomes, and (7) in the selection of reported results.

Each domain was rated as having a low, moderate, serious, or critical risk of bias. The overall study-level risk was determined based on domain-level assessments, and discrepancies were resolved by consensus. The GRADE approach of the Cochrane Collaboration risk of bias (RoB) version 2 assessment tool was used to assess the quality of one randomized controlled study.

#### 2.3.2. Assessment of Consistency and Inconsistency

Consistency between direct and indirect evidence within the network was evaluated using the node-splitting approach, which compares effect estimates derived from direct comparisons with those inferred indirectly through the network. A visual inconsistency plot was generated by comparing posterior mean residual deviance under consistency and inconsistency models. Data points located on the line of equality indicated agreement between models, while those above and below suggested potential consistency and inconsistency within the network [17].

#### 2.3.3. Reporting Quality

The reporting quality of this network meta-analysis was evaluated using the PRISMA-NMA checklist to ensure transparent reporting of methodology, assumptions (including transitivity and similarity), statistical models, and ranking results.

#### 2.3.4. Publication Bias

Publication bias and small-study effects were assessed using funnel plot for direct comparisons, with analyses performed using Review Manager (RevMan) version 5.4.1 [18].

### 2.4. Statistical Analysis

#### 2.4.1. Pairwise Meta-Analysis

Conventional pairwise meta-analyses were conducted using Review Manager (RevMan) version 5.4.1 to estimate pooled effect sizes for direct comparisons at the end of the treatment period. Heterogeneity was assessed using the I^2^ statistic, where a value ≤ 50% was considered indicative of low-to-moderate heterogeneity, and a fixed-effects model was applied; if I^2^ > 50%, a random-effects model was considered. Effect sizes are expressed as odds ratios (ORs) with corresponding 95% confidence intervals (CIs), and statistical significance was defined as *p* < 0.05.

#### 2.4.2. Bayesian Network Meta-Analysis

Bayesian network meta-analysis was performed using WinBUGS version 1.4.3 (MRC Biostatistics Unit, Cambridge, UK), NetMetaXL (version 1.6.1), and MetInsight (version 6.4.2) [19,20]. A Bayesian network was applied to estimate pooled effect sizes and corresponding 95% credible intervals (CrIs) for all treatment comparisons, with both direct and indirect evidence synthesized simultaneously within a consistency model. Inconsistency was evaluated through node-splitting analysis and model fit using posterior mean residual deviance.

#### 2.4.3. Treatment Ranking

Treatment ranking probabilities were estimated using the surface area under the cumulative ranking curve (SUCRA) values, which range from 0% to 100%, with higher values indicating a greater probability that a treatment is among the most effective options [21].

#### 2.4.4. Sensitivity Analysis

Sensitivity analyses were conducted using node-splitting models and by sequentially excluding individual studies to assess the robustness and stability of network estimates.

## 3. Results

### 3.1. Study Selection and Baseline Characteristics

A total of 50 studies were identified through database searches of PubMed, Embase, and the Cochrane Library. After duplicate removal and eligibility screening, 18 studies were ultimately included in the network meta-analysis [22,23,24,25,26,27,28,29,30,31,32,33,34,35,36,37,38,39], in accordance with PRISMA 2020 reporting guidelines [15]. The study selection process is presented in Supplementary Figure S1.

The baseline characteristics of the included studies and patients are summarized in Supplementary Table S2. In fifteen studies, complete baseline demographic and clinical characteristics were reported; in one study, Child–Pugh classification and hepatitis B surface antigen (HBsAg) status were not reported [37]; in two studies, detailed PVTT classification data were not provided [22,25]; and in one study, patients with PVTT were excluded [23].

Regarding treatment comparisons, in five studies, SBRT-based combination regimens versus SBRT, TACE, or immunotherapy monotherapy were evaluated [22,23,27,30,33]; in another five, SBRT monotherapy was compared with TACE, intensity-modulated radiotherapy (IMRT), SBRT plus lenvatinib, or lenvatinib monotherapy [24,25,26,32,34].

**Figure 1:**
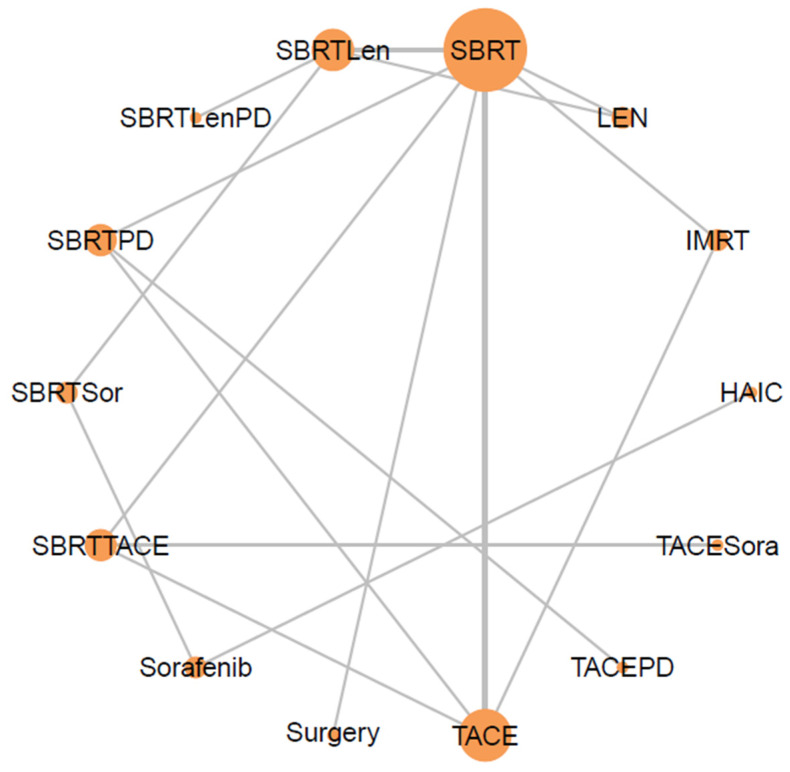
Network evidence plot showed compared treatment regimens for aHCC > 5 cm. The size of a node and the thickness of its edges represent the number of studies examining a particular treatment method and comparing two given treatment methods, respectively.

The geometry of the treatment network is shown in Figure 1. Node size corresponds to the total number of participants receiving each intervention, and edge thickness reflects the number of direct comparisons between treatment pairs.

### 3.2. Quality Assessment Results

#### 3.2.1. Risk of Bias and Publication Bias

Results from the ROBINS-I tool and RoB version 2 indicate that the risk of bias is low to moderate in retrospective cohort studies and in a phase III randomized controlled trial, respectively.

**Figure 2:**
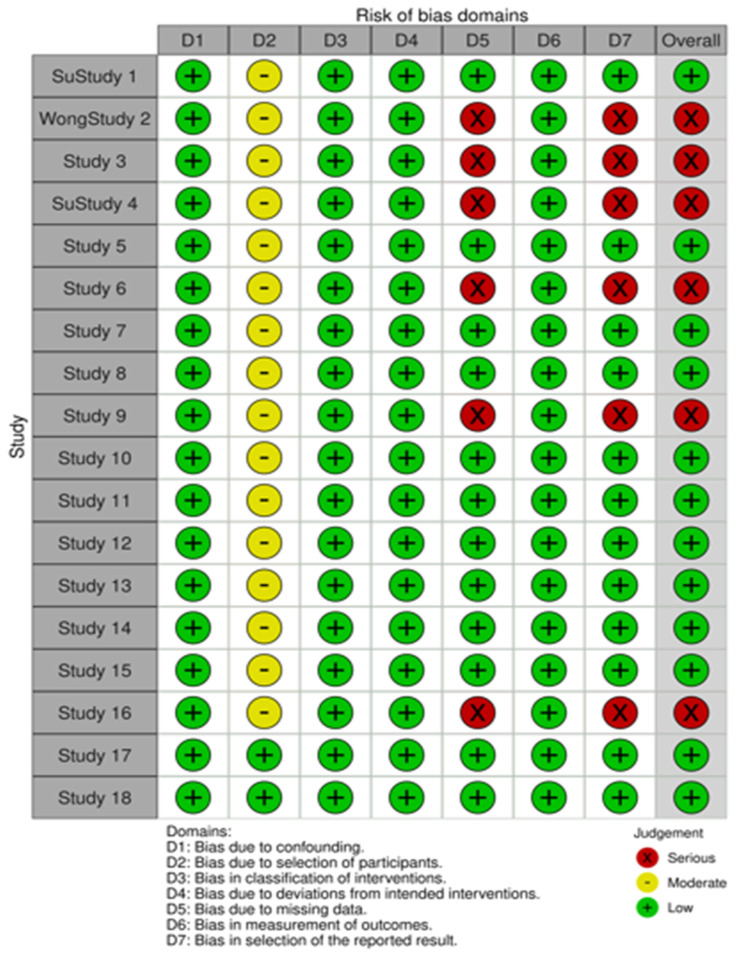
Cochrane risk of bias assessment tool version 2 for randomized trials. Green color represent low risk of bias, yellow and red color represent moderate and serious risk on the related domains, respectively.

Overall, six retrospective cohort studies (35.3%) were judged to have a serious risk of bias, primarily in Domains 5 (missing data) and 7 (selective reporting). Specifically, in two studies, median overall survival (mOS) was not reported; in two studies, progression-free survival (PFS) data were absent; and in two studies, the authors reported that OS and PFS endpoints were not reached at the time of analysis. The remaining 12 studies (64.7%) were assessed as having low risk of bias. The detailed assessment is shown in Figure 2.

Publication bias was evaluated using funnel plots (Supplementary Figure S2), with the distribution of studies generally symmetrical around the vertical line, suggesting a low likelihood of small-study effects. One study (Xiang et al.) [29] showed potential asymmetry.

#### 3.2.2. Heterogeneity and Consistency

In the pairwise direct meta-analyses, no significant statistical heterogeneity was observed for either OS or PFS outcomes (I^2^ = 0%; Supplementary Figure S3).

Node-splitting analysis revealed no significant inconsistency between direct and indirect comparisons (all *p* > 0.05; Supplementary Figure S4). Additionally, posterior mean deviance plots demonstrated that all studies were distributed along the line of equality (Supplementary Figure S5), indicating good agreement between consistency and inconsistency models.

Overall, the network demonstrated acceptable transitivity, low heterogeneity, and no evidence of significant inconsistency.

Sensitivity analyses showed no significant difference between direct and indirect comparisons after excluding studies with high potential for bias; thus, our findings are robust (Supplementary Figure S6).

### 3.3. Network Meta-Analysis of Clinical Outcomes

#### 3.3.1. Overall Survival (OS)

Overall survival data were extracted from 18 studies (17 cohort studies and 1 randomized controlled trial), comprising 2597 patients. Fourteen treatment strategies were compared within the network (Table 1), and in the league table, odds ratios (ORs) < 1 favor the treatment listed in the corresponding row. SBRT combined with TACE (SBRT + TACE) ranked first in terms of improving OS, demonstrated significantly improved OS compared with SBRT plus PD-1 inhibitors (SBRT + PD-1) (OR = 0.26; 95% CI: 0.16–0.69), and was also superior to TACE monotherapy (OR = 0.55; 95% CI: 0.32–0.97).

Interestingly, SBRT monotherapy was statistically superior to SBRT + PD-1 (OR = 0.41; 95% CI: 0.17–0.91), although no significant differences were observed when compared with surgery, sorafenib, lenvatinib, TACE, IMRT, SBRT+ lenvatinib+ PD-1 inhibitors, SBRT plus lenvatinib, or TACE plus PD-1 inhibitors.

TACE combined with sorafenib demonstrated a modest improvement in OS compared with several other regimens, ranking second overall.

**Figure 3:**
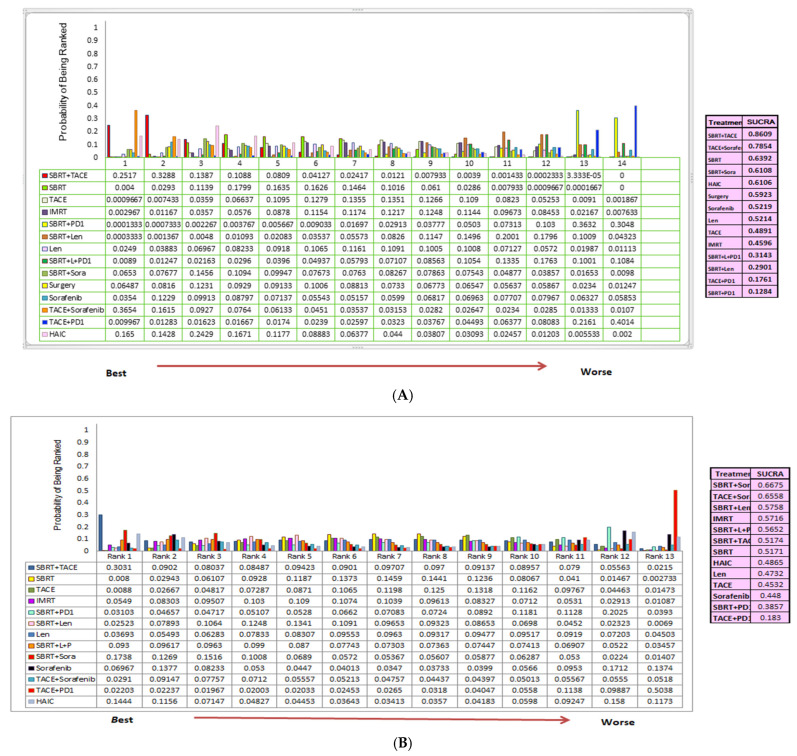
(**A**) Ranking chart of best OS probabilities with different intervention measures and (**B**) best PFS probabilities with different intervention measures.” Annotation. Ranking chart of best OS and PFS with different intervention measures.

Treatment ranking based on SUCRA values (Figure 3A) indicated that SBRT + TACE had the highest probability of being the most effective regimen (32.88%), followed by TACE + sorafenib (6.5%). SBRT + PD-1 ranked lowest in terms of its OS benefit.

#### 3.3.2. Progression-Free Survival (PFS)

A total of two studies lacking PFS data were excluded from this analysis, resulting in 16 studies (15 cohort studies and 1 randomized trial), comprising 2320 patients.

Across the network, no statistically significant differences were observed among treatment regimens for PFS (Table 1).

Based on SUCRA ranking, SBRT + TACE regimen was the most effective in improving OS, with the highest probability of being the best regimen (86.9%), followed by TACE + sorafenib, with a probability of 78.54%.for OS (Figure 3A). SBRT plus sorafenib demonstrated the highest probability (66.75%) of being the most effective treatment for PFS, followed by TACE + sorafenib (65.58%) and SBRT plus lenvatinib (57.58%). TACE plus PD-1 ranked lowest (18.3%) (Figure 3B).

#### 3.3.3. Grade 3–4 Adverse Events

Grade 3–4 adverse event (AE) data were extracted from all 18 studies, comprising 2733 patients and 13 intervention strategies (Table 2). SBRT combined with PD-1 inhibitors demonstrated a significantly lower incidence of severe AEs compared with several monotherapies, including the following:

SBRT (OR = 0.32; 95% CI: 0.11–0.83), IMRT (OR = 0.25; 95% CI: 0.08–0.71), TACE (OR = 0.22; 95% CI: 0.08–0.57), and surgery (OR = 0.16; 95% CI: 0.05–0.54). Compared with other combination regimens, no statistically significant differences were observed with TACE + PD-1 or lenvatinib-containing regimens. The most frequently reported grade 3–4 adverse events included thrombocytopenia, leukopenia, decreased platelet count, elevated alanine aminotransferase (ALT)/aspartate aminotransferase (AST), hypertension, and hyperbilirubinemia.

The sorafenib-containing regimens were associated with a higher incidence of hematologic and hepatic toxicities, including thrombocytopenia (5%), leukopenia (13%), neutropenia (12%), elevated ALT/AST (18%), and hypertension (9%) (Supplementary Table S3).

## 4. Discussion

In this network meta-analysis, we demonstrate that SBRT combined with TACE provides superior overall survival compared with TACE monotherapy and SBRT combined with PD-1 inhibitors in patients with large (≥5 cm) hepatocellular carcinoma complicated by portal vein tumor thrombus (PVTT). These findings reinforce the growing consensus that integrating locoregional and systemic strategies yields improved therapeutic outcomes in advanced HCC [40].

### 4.1. Rationale for the Superiority of SBRT + TACE

Current clinical practice guidelines, including those from the National Comprehensive Cancer Network (NCCN) and the American Association for the Study of Liver Diseases, recommend TACE as the preferred treatment for unresectable HCC; however, in tumors larger than 5 cm, repeated TACE sessions are often required, increasing the risk of cumulative hepatic toxicity and deterioration of liver function [41,42].

The combination of SBRT and TACE offers complementary mechanisms of action: the latter induces tumor necrosis by embolizing tumor-feeding arteries and delivering high concentrations of chemotherapeutic agents locally [43]; the former subsequently delivers ablative radiation doses with high precision, achieving local tumor control while minimizing collateral damage to surrounding liver tissue and delay of systemic therapy resistance. Beyond cytotoxicity, SBRT remodels the tumor microenvironment by inducing immunogenic cell death, promotes tumor-associated antigen release, and enhances dendritic cell activation [44,45]. TACE-induced tumor necrosis may further augment antigen exposure, amplifying immune priming. This dual-modality approach may therefore exert synergistic effects on both local tumor eradication and systemic antitumor immunity.

The authors of several recent clinical studies have reported improved survival with SBRT + TACE compared with either modality alone in large or PVTT-positive HCC. Our findings consolidate these observations within a comparative network analysis, supporting SBRT + TACE as a particularly effective strategy in patients with substantial tumor burden and vascular invasion.

### 4.2. Role of TACE Combined with Targeted or Immune Therapy

TACE combined with multikinase inhibitors such as sorafenib has emerged as another promising strategy. Sorafenib inhibits tumor proliferation through a blockade of RAF kinases in the MAPK pathway and suppresses angiogenesis via inhibition of VEGFR and PDGFR signaling, and can further modulate the immune response by inducing macrophage pyroptosis, thereby triggering natural-killer-cell-mediated cytotoxicity against HCC [46,47]. By counteracting TACE-induced and hypoxia-driven angiogenic rebound, sorafenib may prolong tumor control, a mechanistic synergy that likely explains why TACE + sorafenib ranked second in terms of overall survival in our analysis.

Similarly, TACE combined with immune checkpoint inhibitors represents an evolving therapeutic concept. TACE-induced local tumor necrosis releases tumor-associated antigens into the microenvironment and danger-associated molecular patterns, potentially enhancing T-cell activation. Immune checkpoint inhibitors may then amplify cytotoxic T-cell responses, strengthening systemic immune-mediated tumor destruction [48]. Although promising, this strategy did not surpass SBRT + TACE in terms of survival benefit within the present network meta-analysis.

### 4.3. Immunomodulatory Effects of SBRT and the SBRT + PD-1 Strategy

SBRT exerts profound immunomodulatory effects within the tumor microenvironment (TME). It is a hypofractionated radiation, usually with a dose more than 2.0 Gy/fraction, and it then promotes the release of tumor-associated antigens and damage-associated molecular patterns, enhances antigen presentation, and increases CD8^+^ T-cell infiltration effects [49], which may counteract the immunosuppressive TME characteristic of advanced HCC. The theoretical synergy between SBRT and PD-1 blockade is compelling; radiation may enhance tumor-associated antigen exposure and upregulate PD-L1 expression, thereby sensitizing tumors to immune checkpoint inhibition. However, in this analysis, SBRT + PD-1 did not demonstrate superior overall survival compared with SBRT + TACE, a finding which may reflect limited sample size, heterogeneity in PD-1 agents and sequencing strategies, and the profoundly immunosuppressive milieu associated with large tumors and PVTT.

Importantly, SBRT + PD-1 demonstrated the most favorable safety profile, suggesting that this regimen may be particularly suitable for patients with compromised liver reserve or intolerance to embolization-based therapies.

### 4.4. Emerging Advances in Locoregional Immunomodulation

Advances in precision drug delivery are further enhancing the therapeutic landscape. Transcatheter arterial chemoembolization (DEB-TACE) with small-sized, drug-loaded microspheres can achieve sustained release kinetics and distribution of chemotherapeutic drugs in the tumor microenvironment and can reduce liver-damaging toxicity [47]. In recent preclinical work, authors have explored transarterial embolization combined with localized immunotherapy delivery, including microsphere-based anti-PD-L1 strategies designed to remodel the immunosuppressive microenvironment and enhance CD8^+^ T-cell activation [50].

These developments support the broader hypothesis that integrating locoregional cytotoxicity with immune modulation can overcome resistance mechanisms in advanced HCC. The superior performance of SBRT + TACE in this network meta-analysis aligns with this evolving paradigm of multimodal tumor microenvironment remodeling.

### 4.5. Safety Considerations

Regarding safety, SBRT + PD-1 was associated with fewer grade 3–4 adverse events compared with other regimens. Sorafenib-containing therapies demonstrated higher rates of hematologic and hepatic toxicities; nevertheless, most severe adverse events were clinically manageable.

It should be noted that adverse event reporting was inconsistent across studies and grading criteria were not uniformly specified. Prospective trials with standardized toxicity assessment are therefore warranted.

### 4.6. Strengths and Limitations

This study represents the first comprehensive network meta-analysis comparing SBRT-based treatment strategies in large HCC with PVTT. The absence of significant heterogeneity and inconsistency strengthens the reliability of the comparative estimates. However, several limitations must be acknowledged: most included studies were retrospective; SBRT dose, fractionation schedules, and sequencing with systemic therapies were heterogeneous; and PD-1 inhibitors were grouped without drug-specific analysis. However, the robustness of the sensitivity analysis results can mitigate these limitations. Future well-designed phase III randomized trials are required to validate these findings and optimize treatment sequencing.

## 5. Conclusions

In this network meta-analysis, we indicate that SBRT combined with TACE provides the most favorable overall survival benefit among currently available treatment strategies for patients with large (≥5 cm) unresectable hepatocellular carcinoma with portal vein tumor thrombus. SBRT combined with PD-1 inhibitors offers a superior safety profile and may be appropriate for selected patients.

These findings underscore the importance of multimodal strategies targeting both locoregional tumor control and tumor microenvironment modulation. Prospective randomized clinical trials and mechanistic studies are needed to confirm these results and refine personalized treatment pathways for advanced HCC.

## Figures and Tables

**Table 1:** (**A**) League table for all treatment modalities’ overall survival outcomes (upper part). (**B**) League table for PFS improvement in all treatment regimens (lower part).

(**A**)													
SBRT + TACE													
1.00 (0.36–2.77)	TACE + Sorafenib												
0.65 (0.37–1.12)	0.65 (0.20–2.08)	SBRT											
0.67 (0.14–3.62)	0.68 (0.10–4.82)	1.04 (0.23–5.05)	HAIC										
0.65 (0.18–2.37)	0.66 (0.13–3.47)	1.01 (0.32–3.17)	0.97 (0.35–2.56)	SBRT + Sora									
0.64 (0.25–1.59)	0.64 (0.16–2.53)	0.98 (0.47–2.03)	0.94 (0.17–5.08)	0.96 (0.25–3.84)	Surgery								
0.59 (0.13–2.92)	0.59 (0.09–4.02)	0.91 (0.22–4.10)	0.87 (0.55–1.38)	0.91 (0.38–2.23)	0.94 (0.18–4.94)	Sorafenib							
0.55 (0.32–0.97)	0.57 (0.14–2.24)	0.87 (0.42–1.83)	0.84 (0.18–3.55)	0.86 (0.28–2.54)	0.89 (0.32–2.50)	0.96 (0.22–3.82)	Len						
0.56 (0.31–0.97)	0.56 (0.17–1.79)	0.86 (0.61–1.19)	0.82 (0.17–3.79)	0.85 (0.26–2.82)	0.87 (0.39–1.95)	0.94 (0.20–4.06)	0.98 (0.44–2.20)	TACE					
0.53 (0.27–1.04)	0.53 (0.16–1.81)	0.82 (0.54–1.24)	0.78 (0.15–3.70)	0.81 (0.24–2.74)	0.83 (0.36–1.92)	0.90 (0.19–3.96)	0.93 (0.40–2.16)	0.95 (0.60–1.52)	IMRT				
0.41 (0.14–1.25)	0.41 (0.09–1.90)	0.64 (0.24–1.65)	0.61 (0.14–2.51)	0.63 (0.22–1.76)	0.64 (0.20–2.15)	0.69 (0.17–2.67)	0.73 (0.29–1.79)	0.75 (0.27–2.06)	0.78 (0.27–2.20)	SBRT + L + PD1			
0.42 (0.16–1.06)	0.41 (0.10–1.67)	0.64 (0.30–1.36)	0.61 (0.15–2.19)	0.63 (0.26–1.47)	0.65 (0.23–1.85)	0.70 (0.19–2.35)	0.73 (0.37–1.45)	0.74 (0.32–1.71)	0.78 (0.33–1.86)	1.00 (0.56–1.79)	SBRT + Len		
0.24 (0.06–1.00)	0.24 (0.04–1.38)	0.38 (0.09–1.43)	0.35 (0.04–2.77)	0.37 (0.06–2.18)	0.38 (0.08–1.75)	0.41 (0.05–2.96)	0.43 (0.09–1.97)	0.44 (0.11–1.69)	0.46 (0.11–1.85)	0.59 (0.11–3.13)	0.59 (0.12–2.78)	TACE + PD1	
0.26 (0.10–0.69)	0.26 (0.06–1.09)	0.41 (0.17–0.91)	0.35 (0.06–2.13)	0.37 (0.08–1.61)	0.38 (0.11–1.21)	0.41 (0.07–2.28)	0.43 (0.12–1.37)	0.44 (0.16–1.09)	0.46 (0.16–1.23)	0.59 (0.15–2.25)	0.59 (0.17–1.94)	1.00 (0.38–2.60)	SBRT + PD1
(**B**)													
SBRT + Sora													
1.02 (0.21–5.13)	TACE + Sorafenib												
0.86 (0.39–1.90)	0.84 (0.21–3.39)	SBRT + Len											
0.84 (0.26–2.72)	0.82 (0.24–2.79)	0.97 (0.41–2.30)	IMRT										
0.86 (0.32–2.27)	0.83 (0.19–3.80)	1.00 (0.55–1.78)	1.02 (0.36–2.91)	SBRT + L + PD1									
0.80 (0.24–2.72)	0.78 (0.27–2.20)	0.92 (0.36–2.32)	0.95 (0.49–1.85)	0.93 (0.30–2.80)	SBRT + TACE								
0.80 (0.27–2.38)	0.78 (0.23–2.47)	0.92 (0.44–1.96)	0.94 (0.61–1.46)	0.93 (0.35–2.39)	1.00 (0.57–1.73)	SBRT							
0.75 (0.17–3.23)	0.73 (0.08–6.17)	0.87 (0.16–4.45)	0.89 (0.13–5.69)	0.87 (0.14–5.05)	0.94 (0.14–6.15)	0.95 (0.15–5.76)	HAIC						
0.75 (0.27–2.19)	0.74 (0.18–2.95)	0.88 (0.45–1.76)	0.90 (0.39–2.13)	0.89 (0.36–2.16)	0.95 (0.38–2.41)	0.96 (0.46–2.01)	1.03 (0.17–6.55)	Len					
0.75 (0.24–2.36)	0.73 (0.22–2.33)	0.87 (0.38–1.97)	0.89 (0.55–1.45)	0.87 (0.31–2.39)	0.94 (0.54–1.63)	0.94 (0.66–1.35)	1.00 (0.16–6.53)	0.98 (0.43–2.22)	TACE				
0.71 (0.17–2.83)	0.69 (0.08–5.61)	0.82 (0.16–3.97)	0.84 (0.13–5.14)	0.82 (0.15–4.43)	0.88 (0.14–5.45)	0.89 (0.15–5.11)	0.95 (0.60–1.50)	0.92 (0.15–5.07)	0.94 (0.15–5.64)	Sorafenib			
0.65 (0.18–2.48)	0.64 (0.16–2.55)	0.77 (0.27–2.22)	0.78 (0.33–1.84)	0.76 (0.23–2.56)	0.82 (0.33–2.05)	0.83 (0.39–1.77)	0.88 (0.12–6.66)	0.87 (0.29–2.46)	0.88 (0.40–1.89)	0.93 (0.14–6.60)	SBRT + PD1		
0.41 (0.08–2.10)	0.40 (0.07–2.16)	0.48 (0.12–1.98)	0.49 (0.13–1.75)	0.48 (0.10–2.22)	0.51 (0.14–1.97)	0.52 (0.15–1.78)	0.55 (0.06–5.12)	0.54 (0.13–2.23)	0.55 (0.16–1.88)	0.58 (0.07–5.11)	0.62 (0.24–1.63)	TACE + PD1	

Annotation: Data are presented as odds ratios (ORs) and 95% confidence intervals (CIs) at the end of treatment. OR < 1 represents favored treatments. Each cell in the top left is better. Abbreviations: Len, lenvatinib; PD1, Programmed Cell Death Protein 1 inhibitors; SBRT + L + PD1, SBRT combined with lenvatinib and PD-1 inhibitors.

**Table 2:** League table for severe adverse events.

SBRT + PD1												
0.41 (0.05–2.88)	HAIC											
0.32 (0.11–0.83)	0.79 (0.14–4.84)	SBRT										
0.39 (0.08–1.49)	0.94 (0.08–12.02)	1.23 (0.18–6.70)	TACE + PD1									
0.25 (0.18–0.71)	0.61 (0.10–4.14)	0.78 (0.43–1.41)	0.64 (0.11–4.40)	IMRT								
0.23 (0.05–1.01)	0.56 (0.11–2.91)	0.70 (0.21–2.27)	0.58 (0.07–5.16)	0.90 (0.24–3.40)	Len							
0.22 (0.08–0.57)	0.54 (0.08–3.71)	0.69 (0.38–1.22)	0.56 (0.10–3.72)	0.88 (0.48–1.61)	0.97 (0.26–3.72)	TACE						
0.21 (0.04–0.90)	0.51 (0.14–1.86)	0.64 (0.19–1.99)	0.53 (0.07–4.67)	0.82 (0.22–2.96)	0.90 (0.35–2.38)	0.93 (0.24–3.39)	SBRT + Len					
0.16 (0.05–0.54)	0.40 (0.06–2.85)	0.51 (0.24–1.08)	0.42 (0.07–3.09)	0.65 (0.25–1.70)	0.72 (0.18–2.90)	0.74 (0.29–1.93)	0.79 (0.21–3.28)	Surgery				
0.12 (0.02–0.76)	0.31 (0.06–1.64)	0.39 (0.07–1.82)	0.32 (0.08–3.53)	0.50 (0.08–2.58)	0.55 (0.12–2.29)	0.57 (0.09–2.95)	0.62 (0.19–1.69)	0.76 (0.12–4.17)	SBRT + L + P			
0.13 (0.04–0.39)	0.30 (0.04–2.39)	0.39 (0.17–0.87)	0.32 (0.05–2.31)	0.50 (0.21–1.18)	0.56 (0.13–2.38)	0.57 (0.29–1.10)	0.61 (0.15–2.62)	0.77 (0.26–2.30)	0.12 (0.17–6.57)	SBRT + TACE		
0.07 (0.01–0.46)	0.16 (0.11–0.25)	0.21 (0.03–1.14)	0.17 (0.01–1.98)	0.26 (0.04–1.58)	0.29 (0.06–1.39)	0.30 (0.05–1.83)	0.32 (0.09–1.09)	0.40 (0.06–2.52)	0.53 (0.10–2.82)	0.53 (0.07–3.53)	Sorafenib	
0.06 (0.01–0.35)	0.16 (0.06–0.42)	0.20 (0.04–0.82)	0.17 (0.02–1.67)	0.25 (0.05–1.18)	0.28 (0.08–1.02)	0.29 (0.06–1.35)	0.31 (0.13–0.71)	0.39 (0.07–1.92)	0.51 (0.13–2.12)	0.50 (0.09–2.68)	0.97 (0.39–2.34)	SBRT + Sorafenib

Annotation: Data are presented a odds ratio (OR) rand 95% confidence intervals (CI) at the end of treatment. OR < 1 favor treatments. Each cell in the topic left is less severe adverse event > Grade 3. Abbreviation: Len, lenvatinib; PD1, Programmed Cell Death Protein 1, inhibitors. SBRT + L + P, SBRT combined with lenvatinib and PD-1 inhibitors.

## Data Availability

The data presented in this study are available on request from the corresponding author due to privacy.

## Supplementary Materials

The following supporting information can be downloaded at: https://www.mdpi.com/article/10.3390/cancers18060988/s1, Figure S1: Flow chart for the identification of the eligible clinical studies; Figure S2: Publication bias presented as funnel plot for overall survival (A) and progression free survival (B); Figure S3: I2 test for heterogeneity in direct comparison of regimens (A) overall survival (B) progress free survival in this network analysis; Figure S4: Node-splitting model for consistency and inconsistency between direct and indirect comparisons; Figure S5: Residual deviance from NMA model and UME inconsistency model for all studies; Figure S6: Node-splitting model for sensitivity analysis; Table S1: Search strategy; Table S2: Basic characteristic of included studies; Table S3. ≧Grade 3 severe adverse events.
