# Supplementary material for: Efficacy and Safety of Stereotactic Body Radiation Therapy Modalities for >5 cm Advanced Unresectable Hepatocellular Carcinoma: A Network Meta-Analysis
Source: Cancers (Basel). 2026 Mar 18;18(6):988. doi: 10.3390/cancers18060988 (PMC13025757; doi:10.3390/cancers18060988)
Supplement: Supplementary file 1 [file cancers-18-00988-s001.zip › Supplementary Table S1 Search strategy 1141201.pdf]

Supplementary Table S1. Search strategy in PubMed, EMbase, the Cochrane Library

Search number	Strategy	Results
PubMed		Results
1	("carcinoma, hepatocellular"[MeSH Terms] OR ("carcinoma"[All Fields] AND "hepatocellular"[All Fields]) OR "hepatocellular carcinoma"[All Fields] OR ("hepatocellular"[All Fields] AND "carcinoma"[All Fields])) AND ("neoplasms"[MeSH Terms] OR "neoplasms"[All Fields] OR ("malignant"[All Fields] AND "tumor"[All Fields]) OR "malignant tumor"[All Fields]) AND ("cancer s"[All Fields] OR "cancerated"[All Fields] OR "canceration"[All Fields] OR "cancerization"[All Fields] OR "cancerized"[All Fields] OR "cancerous"[All Fields] OR "neoplasms"[MeSH Terms] OR "neoplasms"[All Fields] OR "cancer"[All Fields] OR ancers"[All.Fields])	128,327
2	"chemoembolization, therapeutic"[MeSH Terms] OR ("chemoembolization"[All Fields] AND "therapeutic"[All Fields]) OR "therapeutic chemoembolization"[All Fields] OR "chemoembolization therapeutic"[All Fields] OR "transcatheter"[All Fields] OR ("transarterial"[All Fields] OR "transarterially"[All Fields]) OR "chemoemboli"[All Fields] OR ("adam17 protein human"[Supplementary Concept] OR "adam17 protein human"[All Fields] OR "tace"[All Fields])	63,082
3	("drug"[All Fields] AND ("elutable"[All Fields] OR "elutant"[All Fields] OR "elute"[All Fields] OR "eluted"[All Fields] OR "elutent"[All Fields] OR "eluter"[All Fields] OR "eluters"[All Fields] OR "elutes"[All Fields] OR "eluting"[All Fields] OR "elution"[All Fields] OR "elutions"[All Fields]) AND ("bead s"[All Fields] OR "beads"[All Fields]) AND ("adam17 protein human"[Supplementary Concept] OR "adam17 protein human"[All Fields] OR "tace"[All Fields])) OR (("drug effects"[MeSH Subheading] OR ("drug"[All Fields] AND "effects"[All Fields]) OR "drug effects"[All Fields] OR "de"[All Fields]) AND ("adam17 protein human"[Supplementary Concept] OR "adam17 protein human"[All Fields] OR "tace"[All Fields]))	2370
4	"SBRT"[All Fields] OR (("radiosurgery"[MeSH Terms] OR "radiosurgery"[All Fields] OR ("stereotactic"[All Fields] AND "body"[All Fields] AND "radiotherapy"[All Fields]) OR "stereotactic body radiotherapy"[All Fields]) AND ("immunotherapy"[MeSH Terms] OR "immunotherapy"[All Fields] OR "immunotherapies"[All Fields] OR "immunotherapy s"[All Fields]))	9203
5	(((("retrospective studies"[MeSH Terms] OR ("retrospective"[All Fields] AND "studies"[All Fields]) OR "retrospective studies"[All Fields] OR "retrospective"[All Fields] OR "retrospectively"[All Fields] OR "retrospectives"[All Fields]) AND ("clinical study"[Publication Type] OR "clinical studies as topic"[MeSH Terms] OR "clinical study"[All Fields])) OR ("clinical study"[Publication Type] OR "clinical studies as topic"[MeSH Terms] OR "clinical study"[All Fields])) AND "English"[All Fields] AND ("human s"[All Fields] OR "humans"[MeSH Terms] OR "humans"[All Fields] OR "human"[All Fields])	15625
6	#1+#2#3#4#5	16

Search number	Strategy	Results
Embase		
1	('unresectable hepatocellular carcinoma'/exp OR 'unresectable hepatocellular carcinoma' OR 'liver cancer'/exp OR 'liver cancer') AND ([controlled clinical trial]/lim OR [randomized controlled trial]/lim) AND [2023-2025]/py	1848
2	(transcatheter:ti,ab,kw OR transarterial:ti,ab,kw) AND chemoemboli*:ti,ab,kw	16886
3	#1AND#2	183
4	sbrt OR stereotactic:ti,ab,kw	72053
5	(sbrt OR stereotactic:ti,ab,kw) AND (lenvatinib:ti,ab,kw OR 'pd 1') AND inhibitors	335
6	#1 AND #4	64
7	(lenvatinib:ti,ab,kw OR 'pd-1') AND inhibitor:ti,ab,kw	21995
8	(#6 OR #2) AND #7	253

Cochrane		
Search number	Strategy	Results
1. unresectable hepatocellular carcinoma with tumor larger than 5 cm		15
2.(transcatheter:ti,ab,kw OR transarterial:ti,ab,kw) AND chemoemboli*:ti,ab,kw		1375
3.(lenvatinib:ti,ab,kw OR 'pdl 1') AND inhibitor:ti,ab,kw		1408
4. (sbrt OR stereotactic:ti,ab,kw)		3175
5. #1 AND #4		1
6. #1 AND #2		12
7.#6 OR #5 AND #3		13
