# Supplementary material for: Efficacy and Safety of Stereotactic Body Radiation Therapy Modalities for >5 cm Advanced Unresectable Hepatocellular Carcinoma: A Network Meta-Analysis
Source: Cancers (Basel). 2026 Mar 18;18(6):988. doi: 10.3390/cancers18060988 (PMC13025757; doi:10.3390/cancers18060988)
Supplement: Supplementary file 1 [file cancers-18-00988-s001.zip › Supplementary Table S2.pdf]

Supplementary Table S2. Basic characteristic of included studies

	Author	Study Design	Patient No. (Groups)		Age (years)	Inclusion criteria		HBsAg + (%)	Cirrhosis (%)	Outcomes, median (months)	
			Intervention (n)	Control (n)		Tumor size, cm	Child-Pugh (A/B)%			OS	PFS
1	Su et al. 2016	Retrospective cohort	SBRT+TACE (n=77)	SBRT(n=50)	51(21-86)	5-10<br>≥10	70/7<br>41/9	73 (94.8)<br>44 (88.0)	57(74)<br>38 (76)	42<br>21	11<br>15
2	Wong et al. 2019	Retrospective cohort	SBRT+TACE(n=49)	TACE (n=98)	61 (28–87)<br>65 (20–90)	9.5 (4–23.6)<br>10.1 (1.8–22.4)	A: 49 (93.9)/85 (86.7)	39 (79.6)<br>77 (78.6)	40 (81.2%)<br>190 (93.9)	23.9<br>10.4	7.6<br>5.7
3	Shen et al. 2019	Retrospective cohort	SBRT (n=34)	TACE (n=68)	62.97(±14.39)<br>63.6 (±11.97)	4.99±1.39<br>4.84±1.486	40/6<br>111/30	23 (50)<br>62 (43.7)	NA	37.1<br>12.6	NA
4	Su et al. 2020	Retrospective cohort	SBRT (n=167)	TACE (n=159)	56 (47, 65)<br>52 (44, 61)	3.4 (2.4, 5.2)<br>7.2 (4.2, 2.1	137 (82.0%)/30 (18.0%)<br>139 (87.4%)/20 (12.6%)	145 (86.8%)<br>141 (88.7%)	NA	71.1<br>37.0	25.3<br>11.3
5	LI et al 2021	Retrospective cohort	SBRT=154	IMRT=133	47 (40, 55)<br>51 (41, 58)	9 (6.3, 11.2)<br>8.1 (5.4, 11)	139 /15<br>115/18	131 (85.1%)<br>110 (82.7%)	NA	10<br>10	6<br>6
6	Chiang et al 2021	Retrospective cohort	SBRT+PD1=16	TACE=48	73 (49–87)<br>66.5(38–86)	10 (3.4–18)<br>10.4(2.68–19.6	14 (87.5)<br>46 (95.8)	12 (75.0)<br>26 (54.2)	NA	23.48<br>7.4	11.7<br>4.83
7	Zhang et al 2022	Retrospective cohort	TACE+Sorafenib=32	TACE+SBRT=30	51.0(40.5-61.0)<br>52.0 (43.25, 59.25	7.85 (6.10, 11.63)<br>9.00 (7.00, 13.28))	27 (84.4) /5 (15.6)<br>29 (96.7)/1 (3.3)	29 (90.6)<br>29 (96.7)	29 (90.6)<br>22 (73.3	8.8<br>13.8	6.3<br>10.4
8	Xiang et al 2022	Retrospective cohort	TACE+PD1=45	SBRT+PD1=31	< 65/≥ 65	4.8 (1.3-12)<br>4.3 (1.6-6)	44/1<br>31/0	40 (82.8)<br>26 (78.9)	38 (84.4)<br>25 (80.6)	14.1<br>11.7	10.1<br>19.5
9	Chiang et al 2023	Retrospective cohort	SBRT+PD1 =25	SBRT=50	72 (38–91)<br>70 (52–88)	9 (3.5–18)<br>6.7 (2.6–17.9)	A:21(70)/ (45 (64.3)	20 (66.7)<br>50 (71.4)	NA	31.6<br>26.3	30.8<br>15.0
10	Ji et al 2023	Retrospective study	SBRT+LEN=37	Len=77	≧55<br><55	≥ 5 cm- <10cm<br>≥ 10 cm	31 (83.8%)/6 (16.2%%)<br>65 (84.4%)/12 (15.6%)	34 (91.9%)<br>73 (94.8%)	NA	19.3<br>11.2	10.3<br>5.3
11	Wang Q et al 2023	Retrospective study	SBRT=35	SBRT+LEN=35	< 60 years<br>≥ 60 years	8.7 (4.8–15.7)<br>7.7 (2.1–15.8)	33 (94.3)/2 (5.7)<br>33 (94.3)/33 (94.3)	32 (91.4)<br>31 (88.6)	35 (100.0)<br>34 (97.1)	11.0<br>16.8	3.7<br>9.1
12	Wang Q et al 2024	Retrospective study	SBRT+L+P=146	SBRT+LEN=68	56.4±9.6<br>56.3±10.8	7.9±4.0<br>8.0±3.9	135(92.5%)/11(7.5%)<br>63(92.6%)/5(7.4%)	129(88.4%)<br>60(88.2%)	125(85.6%)<br>62(91.2%)	31.2<br>17.4	15.6<br>8.8
13	Ji X et al. 2024	Retrospective study	SBRT=38	Len=38	< 60 years<br>≥ 60 years	≥ 5～≥ 10	36 (94.7)/2 (5.3)<br>34 (89.5)/4 (10.5)	36 (94.7)<br>37 (94.7)	38 (100)<br>38 (100)	14.5<br>11.1	6.8<br>5.0
14	Zhang et al. 2024	Retrospective study	SBRT+Len=48	SBRT+Sira=55	56.83 ± 8.059<br>58.67 ± 7.720	≥ 8	32 (66.7%)/16 (33.3%)<br>32 (58.2%)/23 (41.8%)	B: 33 (68.8%)/<br>33 (60.0%)	NA	14.4<br>8.3	12.3<br>7.3
15	Huang et al 2025	Retrospective study	TACE=48	IMRT=60	< 60 years<br>≥ 60 years	<10<br>≥ 10	44 (91.7%)/4 (8.3%)<br>56 (93.3%)/4 (6.7%)	44 (91.7%)<br>55 (91.7%)	39 (81.2%)<br>50 (83.3%)	8.92<br>19.31	4.72<br>10.66
16	Yan et al 2025	Retrospective study	Surgery=38	SBRT=137	< 60 years<br>≥ 60 years	≥ 5<br><5	NA	NA	29 (76.32)<br>122 (89.05	24<br>13	NA
17	Dawson et al 2025	RCT, Phase III	Sorafenib=92	SBRT+sorafenib=85	67 (60.5--75)<br>66 (60-70)	8.2 (1.0-19.1)<br>7.5 (1.0-18.8)	69 (75)/ 23 (25)<br>64 (75)/ 21 (25)	17 (18)/16 (19)	NA	12.3<br>15.8	5.5<br>9.2
18	Lyu 2018	Retrospective study	Sorafenib=147	HAIC=147	50 (16-82)<br>52 (25-77)	11.7± 3.8<br>11.7 ±3.9	98 (66.7)/49 (33.3)<br>94 (63.9)/94 (63.9)	130 (88.4)<br>128 (87.1)	100 (68)<br>104 (70.7)	7.0<br>14.5	3.6<br>7.4

Annotation: TACE or TAE, transarterial hepatic embolization ; TARE,transarterial radioembolization; TACEHL: TACEor DEB-TACE +HAIC+Len (Lenvatinib);
