# Supplementary material for: Efficacy and Safety of Stereotactic Body Radiation Therapy Modalities for >5 cm Advanced Unresectable Hepatocellular Carcinoma: A Network Meta-Analysis
Source: Cancers (Basel). 2026 Mar 18;18(6):988. doi: 10.3390/cancers18060988 (PMC13025757; doi:10.3390/cancers18060988)
Supplement: Supplementary file 1 [file cancers-18-00988-s001.zip › Supplementary Table S3. SBRT Grade 3 ADE comparison 1150215.pdf]

Table S3.≧Grade 3 severe adverse events

Authors Study	Intervention Comparator	Total patients no ≧ 3%	Hematology ,no (%)						Non-Hematology ,no (%)								
			Thrombo-cytopenia	Leucopenia	Anemia	↓ Platelet	WBC ↓	HgB ↓	Vomiting nausea	Diarrhea	ALT/AST increased	↑ Bilirubin	RILD/ Liver F	RI-GI bleeding	HTN	Others	
Su et al 2016	TACE+SBRT<br>SBRT	7/77 (9)<br>5/50 (0.1)	0	0	0	0	0	0	2 (3%)<br>1 (1%)	0	0	0	2 (3)<br>1 (1)	NA	0	3 (4)<br>3 (4)	
Wong T et al 2019	TACE+SBRT<br>TACE	23/49 (47)<br>27 /98 (28)	0	0	0	4 (8.2)<br>3 (3.1)	1 (2)<br>0 (0)	3 (6.1)<br>0 (0.0)	0	0	9 (18.4)<br>14 (14.4)	6 (12.2)<br>7 (7.2)	NA	NA	0	0 (0)<br>3(3.1)	
Shen et al 2019	SBRT<br>TACE	9/46<br>26/142	0	0	0	0	0	0	0	0	0	0	9 (19.6)<br>26 (18.3)	NA	0	0	
Su et al 2020	SBRT<br>TACE	2/167 (1.2)<br>2/159(1.3)	0	0	0	0	0	0	0	0	0	0	2<br>2	NA	0	0	
Li et al 2021	SBRT<br>IMRT	16/154(10.4%)<br>18/133(13.5)	0	0	0	0	0	0	0	0	0	0	12(11.8)<br>15(14.7)	4 (3)<br>3 (2.3)	0	NA	
Chiang et al 2021	SBRT+PD1<br>TACE	3/16 (18.8)<br>42/48(87.5)	0	0	0	1 (6.3)<br>6 (12.5)	0	0 (0.0)<br>4 (8.3)	0	0	0 (0.0)<br>25 (52.1)	1(6.3)<br>5(10.4)	NA	NA	0	1(6.3)<br>2(4.2)	
Xiang et al 2022	TACE+PD<br>SBRT+PD1	9/45 (20)<br>3/31 (10)	0	0	0	3 (6.7)<br>0 (0.0)	0	0	0	0	4 (4.4)<br>2 (3.2)	0	0	0	0	2(4.4)<br>1(3.2)	Hand-foot
Chiang et al 2023	SBRT+PD1<br>SBRT	7/25 (28)<br>9 /50(18)	0	0	0	0 (0.0)<br>4 (10.0)	0	0	0	0	4 (20)<br>2 (5)	1 (5)<br>1 (2.5)	NA	2(5)<br>2 (2.5)	0	2(8)<br>2(4)	
Ji et al 2023	SBRT+Len<br>Len	7 (18.9%)<br>12 (15.6%)	0	0	0	0	0	0	1 (2.7)<br>3 (3.9)	1 (2.7)<br>1 (1.3)	1 (2.7)<br>0 (0)	0	0	0	2 (5.4)<br>6 (7.8)	2(5.4)<br>2(2.6)	
Wang Q et al 2023	SBRT+Len<br>SBRT	5/35 (14.3)<br>4/35 (11.4)	0	0	0	0	0	0	1(2.9)<br>1 (2.9)	1 (2.9)<br>0 (0.0)	0	0	2 (5.7)<br>3 (8.6)	0	0	1(2.9)<br>0 (0.0)	
Wang 2024	SBRT+L+P<br>SBRT+Len	15/146<br>5/68	2(1.4)<br>1(1.5)	3(2.1)<br>1(1.5)						1(0.7)<br>0 (0.0)	5(3.4)<br>1(1.5)				2(1.4)<br>1(1.5)	3(2.1)<br>1(1.5)	
JiX et al 2024	SBRT<br>Len	3/38 (7.9)<br>5/38 (13.2)	2(5.3)<br>0(0.0)	0	0	0	0	0	0 (0)<br>1 (2.6)	0	0(0.0)<br>1 (2.6)	0	1(2.6)<br>0(0.0)	0	0 (0.0)<br>2 (5.3)	0 (0.0)<br>1 (2.6)	
Zhang et al 2022	Sorafenib+TACE<br>SBRT+TACE	0/32 (0)<br>2/30 (6.7)	0	0	0	0	0	0	0	0	0	0	0 (0)<br>2 (6.7)	0	0	0	
Zhang et al, 2024	SBRT+Len<br>SBRT+Sorafenib	23/48 (47.9)<br>38/55 (69)	0	0	3(6.3)<br>3(5.5)	2 (4.2)<br>10(18.2)	2 (4.2)<br>6 (10.9)	0	3 (6.3)<br>2 (3.6)	1 (2.0)<br>2 (3.7)	5 (10.4)<br>6 (10.9)	1 (2.0)<br>2 (3.7)	NA	1(2.0)<br>0 (0.0)	0	5 (10.4)<br>7 (13)	
Huang et al 2025	TACE<br>IMRT	23/48 47.9()<br>23/60 (43)	4 (8.3%)<br>4 (6.7%)	4 (8.3%)<br>5 (8.3%)	0	0	0	0	5 (10.4)<br>3 (5)	1 (2)<br>1 ((1.7)	6(12.5)<br>3 (5)	4 (8.3)<br>1 (1.7)	NA	NA	6 (12.5)<br>6 (10.0)	2 (3.4)<br>3 (5.0)	
Yan et al 2025	Surgery<br>SBRT	17/38 (45)<br>40/137 (29)	0	0	0	0	0	0	0	0	0	0	17 (45)<br>37 (27)	0 (0.0)<br>3 (2.0)	0	13(34)<br>0 (0.0)	
Dawson et al 2025	Sorafenib<br>SBRT+Sorafenib	72/88 (82)<br>66/83 (80)	0	0	0	5(6)<br>9 (11)	0	0	4 (4.5)<br>6 (7.5)	5 (6)<br>2 (2)	16 (18)<br>14 (17)	10 (11)<br>15 (18)	5 (5.6)<br>2 (2.4)	4 (5)<br>4 (5)	11 (13)<br>9 (11)	16 (18)<br>11 (13)	
Lyu et al 2018	Sorafebib<br>HAIC	1136/232 (68.9)<br>62/180 (34.4)	11 (5)<br>6(3)	29(13)<br>15(8)<br>Neutropenia<br>28(12)/10(6)	0	0	0	9(4)<br>4(2)	5 (2)<br>12 (21)	29（3）<br>0	22+21(18.5)<br>9(15.9) AST	0	0	0	22 (9)<br>0	12(5)/0<br>11 (5)/5(3)	Fatique<br>Hypoalbu<br>minemia
