# Supplementary material for: Efficacy and Safety of Stereotactic Body Radiation Therapy Modalities for >5 cm Advanced Unresectable Hepatocellular Carcinoma: A Network Meta-Analysis
Source: Cancers (Basel). 2026 Mar 18;18(6):988. doi: 10.3390/cancers18060988 (PMC13025757; doi:10.3390/cancers18060988)
Supplement: Supplementary file 1 [file cancers-18-00988-s001.zip › Supplementary Figure S1-S6 1150318.pptx]

## Slide 1
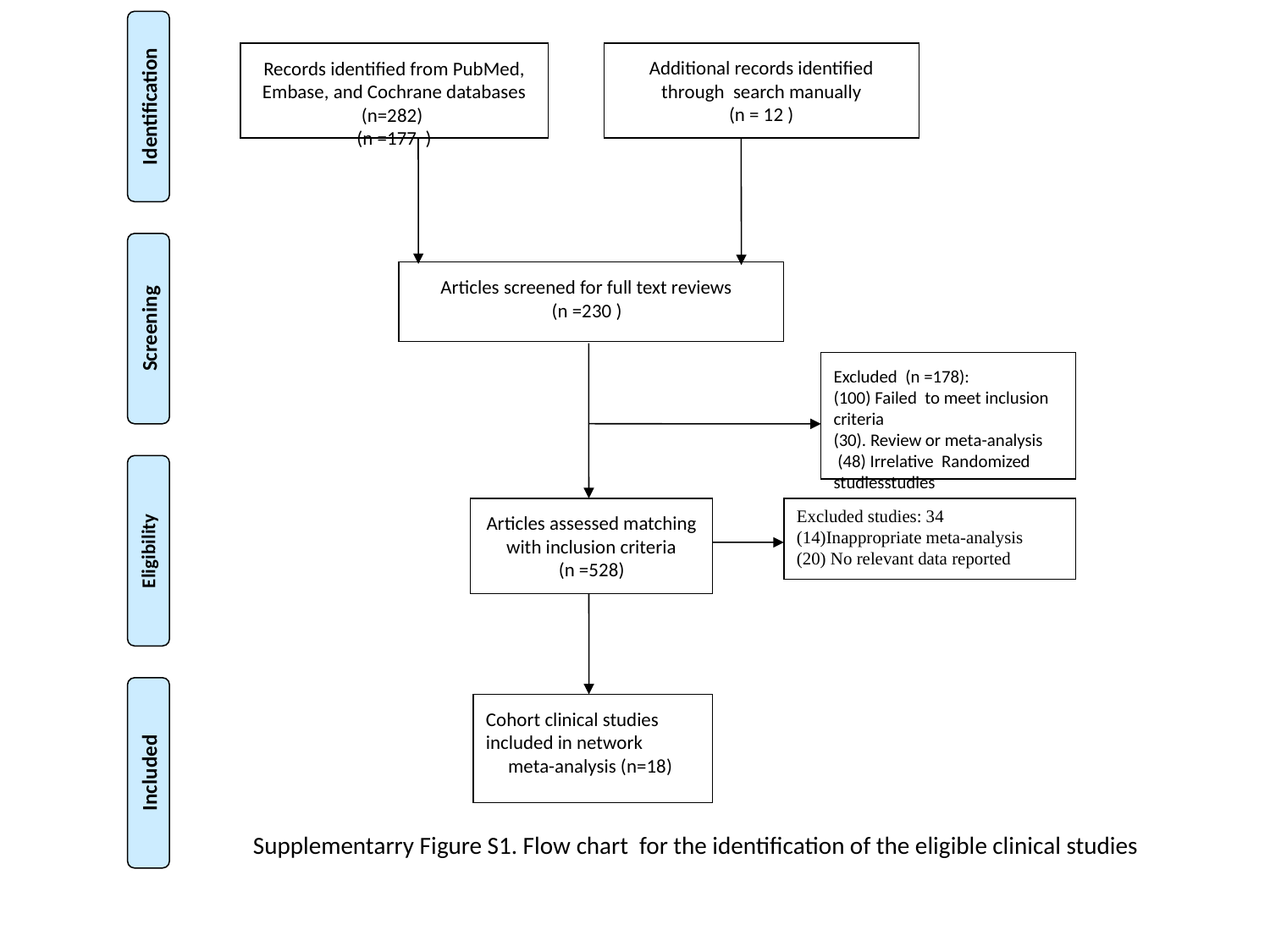

Additional records identified through search manually
(n = 12 )
Records identified from PubMed,
Embase, and Cochrane databases (n=282)
(n =177 )
Identification
Articles screened for full text reviews
 (n =230 )
Screening
Excluded (n =178):
(100) Failed to meet inclusion criteria
(30). Review or meta-analysis
 (48) Irrelative Randomized studiesstudies
Articles assessed matching with inclusion criteria
(n =528)
Excluded studies: 34
(14)Inappropriate meta-analysis
(20) No relevant data reported
Eligibility
Cohort clinical studies included in network
 meta-analysis (n=18)
Included
Supplementarry Figure S1. Flow chart for the identification of the eligible clinical studies
Supplementarry Figure S1. Flow chart for the identification of the eligible clinical studies

## Slide 2
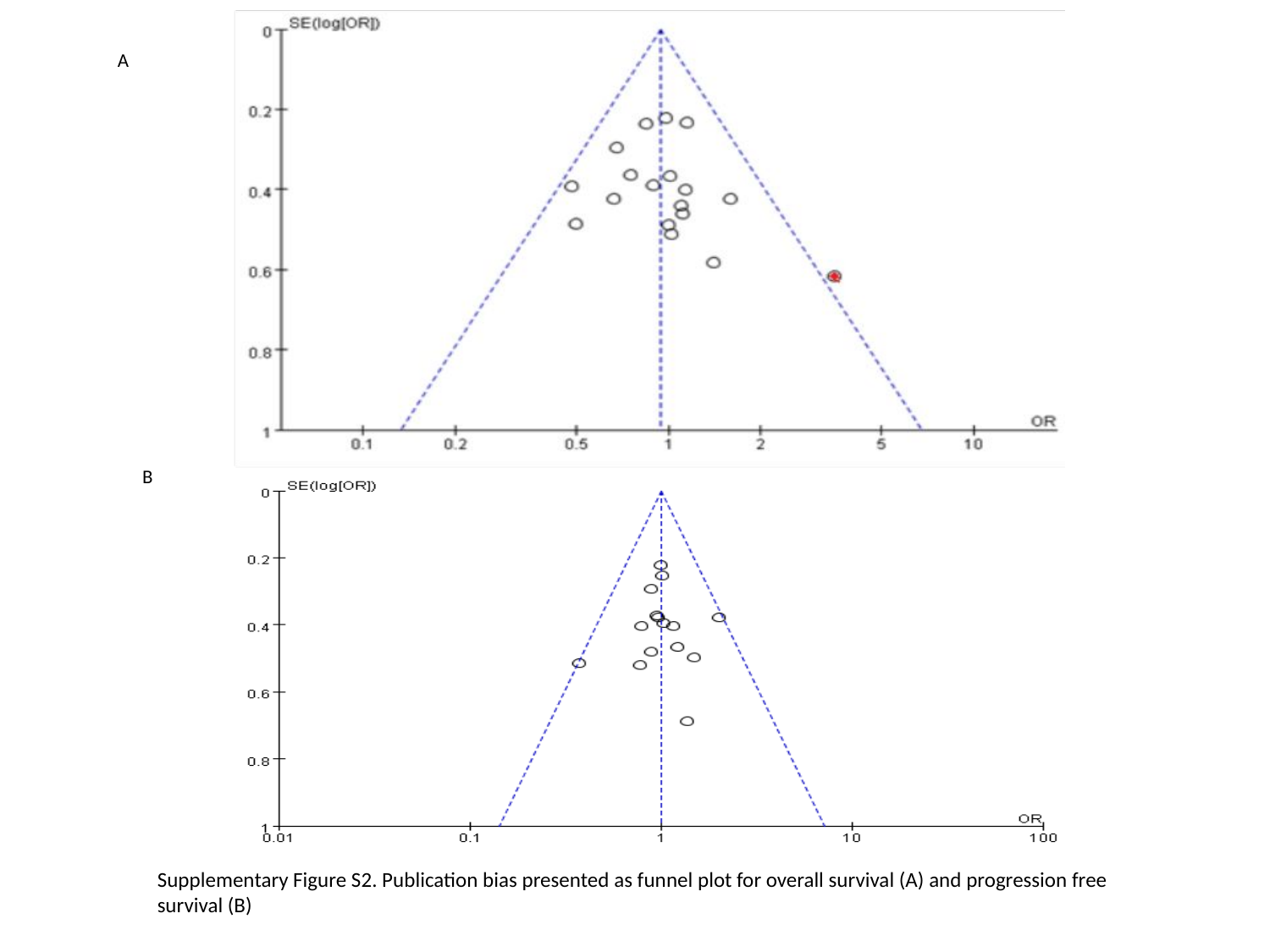

A
B
Supplementary Figure S2. Publication bias presented as funnel plot for overall survival (A) and progression free survival (B)

## Slide 3
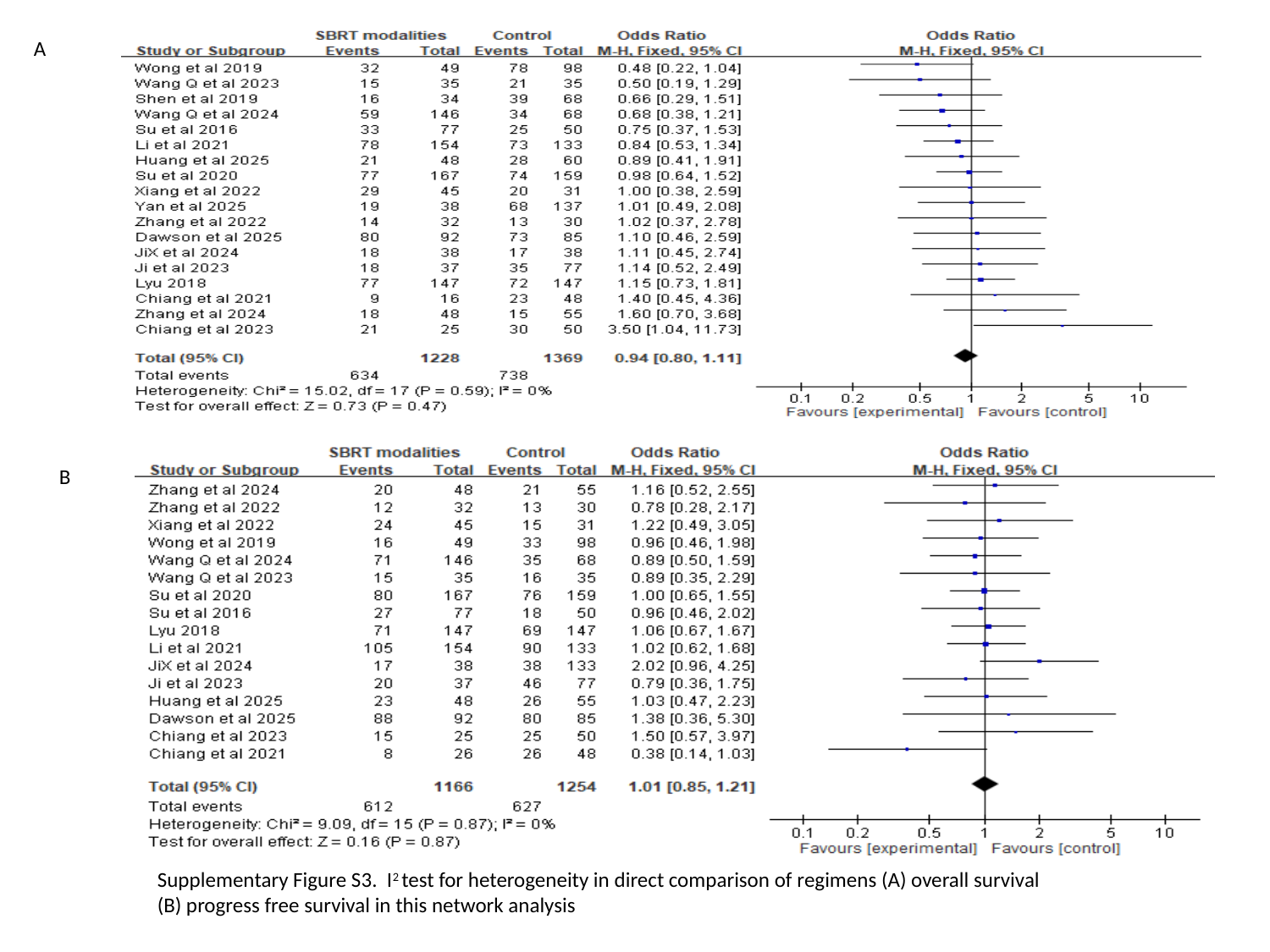

A
B
Supplementary Figure S3. I2 test for heterogeneity in direct comparison of regimens (A) overall survival
(B) progress free survival in this network analysis

## Slide 4
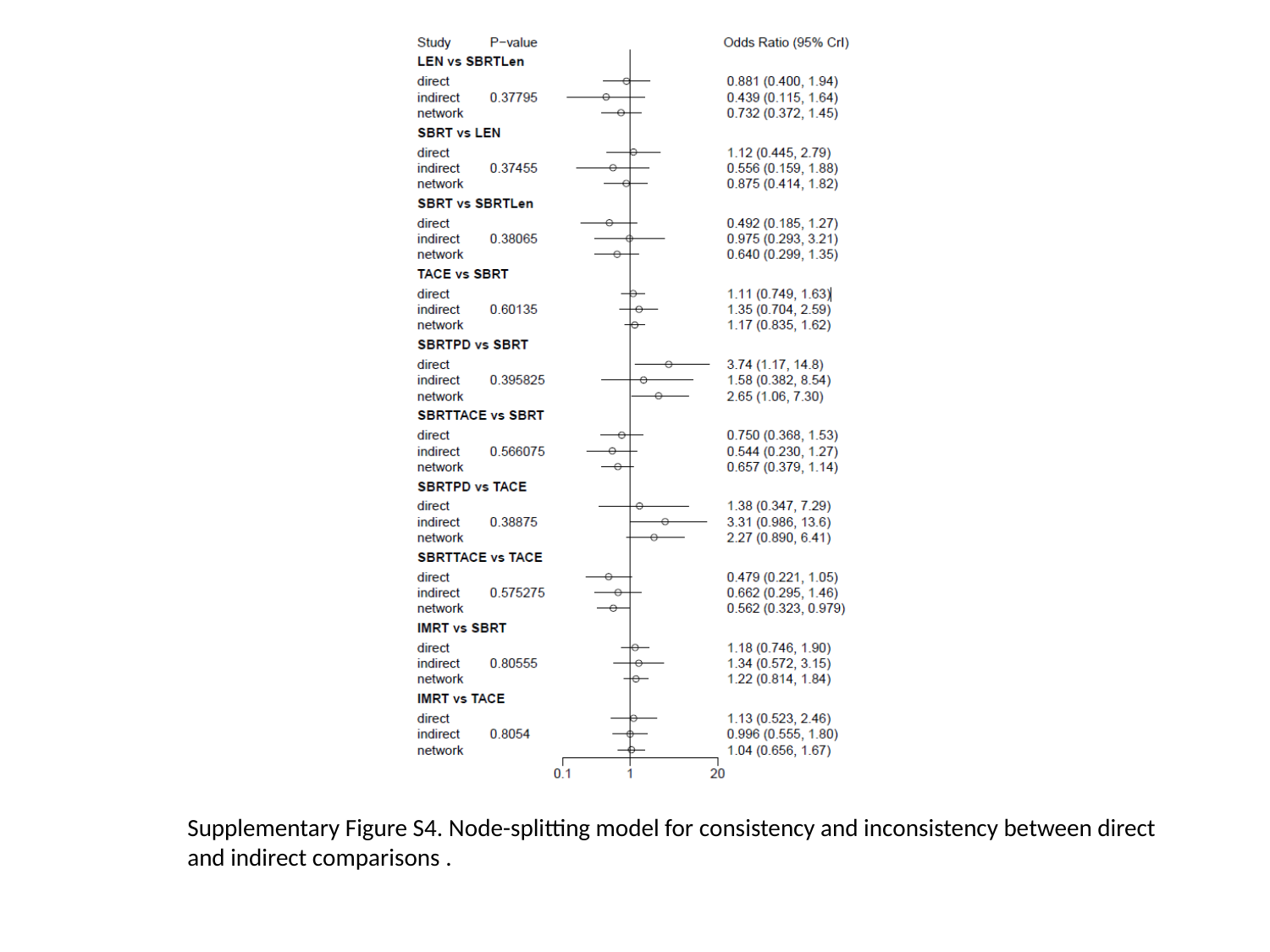

Supplementary Figure S4. Node-splitting model for consistency and inconsistency between direct and indirect comparisons .

## Slide 5
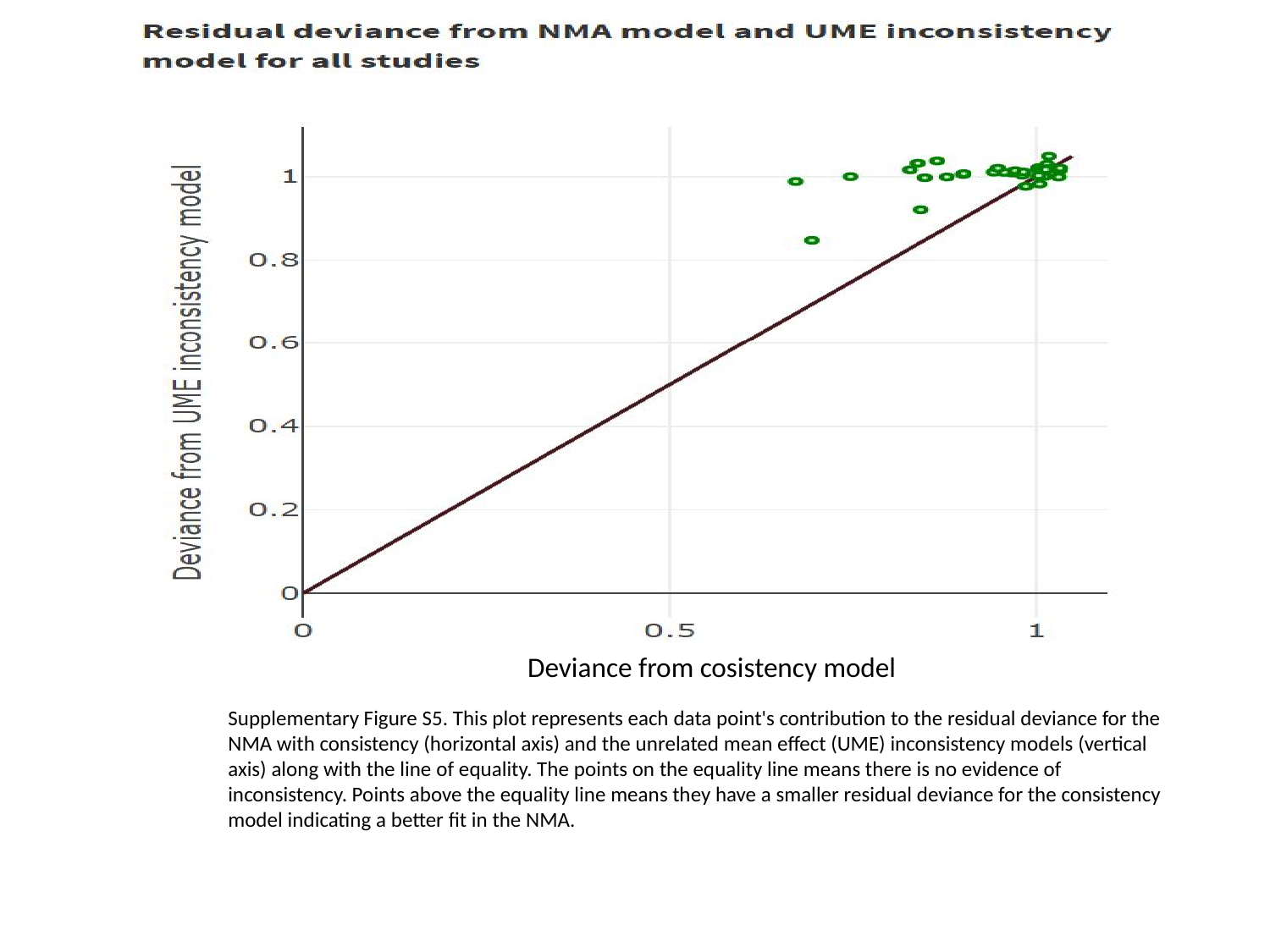

Deviance from cosistency model
Supplementary Figure S5. This plot represents each data point's contribution to the residual deviance for the NMA with consistency (horizontal axis) and the unrelated mean effect (UME) inconsistency models (vertical axis) along with the line of equality. The points on the equality line means there is no evidence of inconsistency. Points above the equality line means they have a smaller residual deviance for the consistency model indicating a better fit in the NMA.

## Slide 6
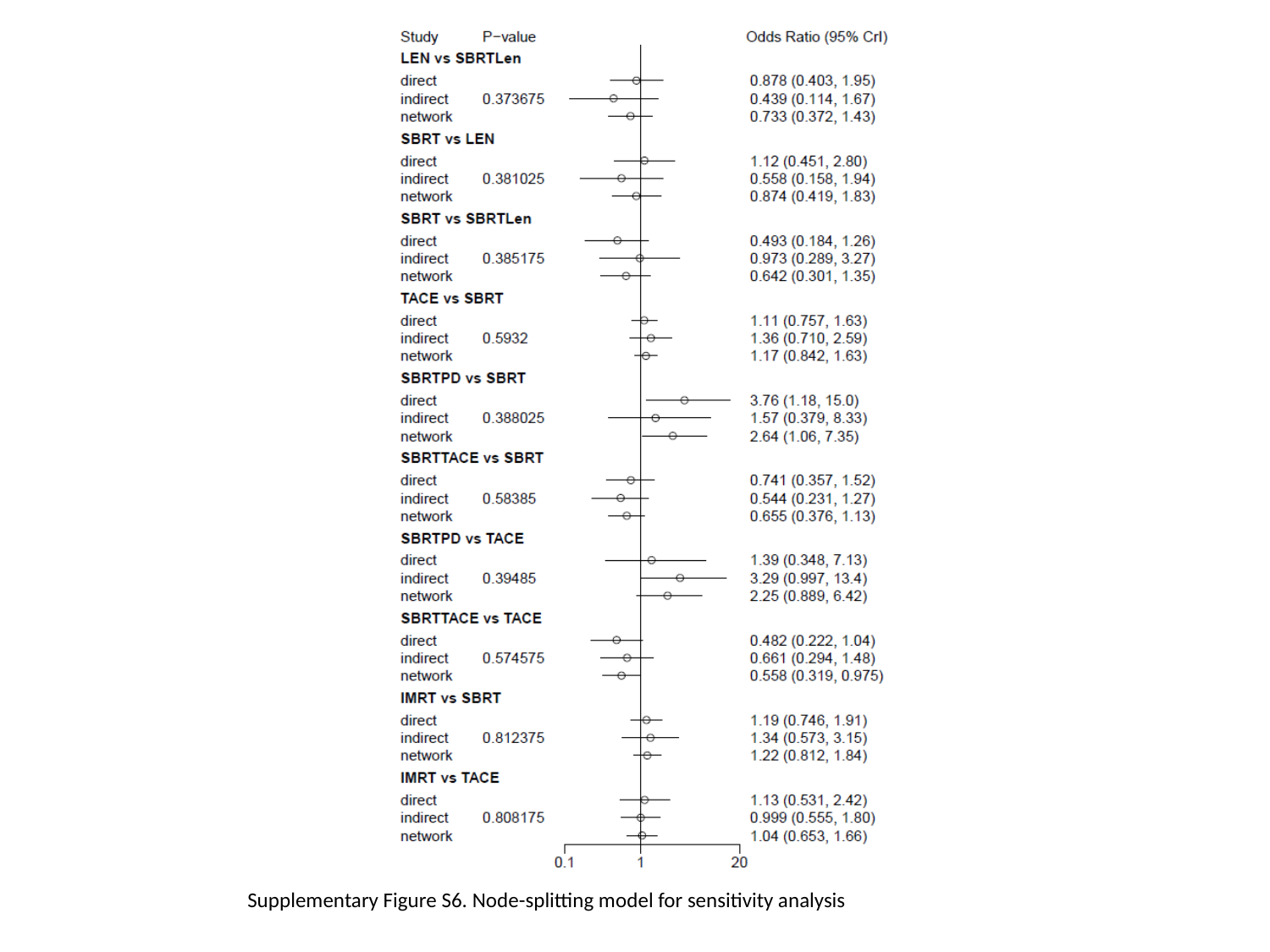

Supplementary Figure S6. Node-splitting model for sensitivity analysis
